# Quality by Design (QbD)-Driven Development and Optimization of Tacrolimus-Loaded Microemulsion for the Treatment of Skin Inflammation

**DOI:** 10.3390/pharmaceutics16121487

**Published:** 2024-11-21

**Authors:** Sanjida Ahmed Srishti, Paromita Paul Pinky, Ryan Taylor, Jacob Guess, Natasha Karlik, Jelena M. Janjic

**Affiliations:** School of Pharmacy, Graduate School of Pharmaceutical Sciences, Duquesne University, Pittsburgh, PA 15282, USA

**Keywords:** quality by design (QbD), microemulsion, design of experiments (DoE), tacrolimus

## Abstract

**Background:** Skin inflammation represents a hallmark of many skin conditions, from psoriasis to eczema. Here, we present a novel microemulsion formulation for delivering a low dose of potent immunosuppressant, tacrolimus, to the skin for local inflammation control. The efficacy of topically delivered tacrolimus in controlling skin inflammation can be enhanced by packaging it into microemulsions. Microemulsions are small-size, thermodynamically stable, and surfactant-rich emulsions that can enhance tissue penetration and local tissue retention of poorly soluble drugs, which can reduce dosing frequency and potentially improve patient compliance. **Methods:** We present a novel approach for microemulsion manufacturing that uses a combination of both low and high-energy methods. The microemulsion composition and manufacturing parameters were optimized by adopting Quality by Design methodologies. The FMECA (Failure, Mode, Effects, Criticality Analysis)-based risk assessment, D-optimal Design of Experiment (DoE), and statistical analysis of parameters impacting responses through the multiple linear regression (MLR) was implemented for identifying critical formulation and process parameters. **Results:** Through QbD strategy, a stable microemulsion with optimized drug loading that met all critical quality attributes (CQAs) was identified. The optimal microemulsion candidate was successfully scaled up three-fold with retained CQAs. The presented microemulsion showed a slow and extended drug release profile in vitro. **Conclusions:** Presented findings suggest that microemulsions are a promising novel approach for tacrolimus delivery to the skin. Further, we also demonstrated that a combination of low-energy emulsification and microfluidization processes can produce stable and robust microemulsions with small droplet size that can be implemented in drug delivery of poorly soluble anti-inflammatory drugs. To the best of our knowledge, this is the first report of QbD-driven optimization of microemulsion manufacturing by microfluidization.

## 1. Introduction

Inflammatory skin conditions, such as psoriasis, atopic dermatitis, and vitiligo, significantly impact the quality of life for many people globally [1,2,3]. Molecular and cellular mechanisms of skin inflammation are complex and involve both the adaptive and innate immune response. When the body is exposed to external stimuli, the skin serves as a first line of defense, supported by the immune response. When this response is dysregulated, the skin inflammation rises, driving the symptoms of skin diseases [4]. T cells, a key component in the adaptive system, are actively involved in the pathogenesis of inflammatory skin conditions. The dysregulation of the immune system or any environmental trigger leads to the distinctive subpopulation of T cells producing specific pro-inflammatory cytokines like interleukins (IL) or Tumor Necrosis Factor-alpha (TNF-α). When the immune system becomes dysregulated, it disrupts healthy cells and causes inflammation [5]. For instance, inflammatory cytokines, such as IL4, IL5, IL13, IL17, and TNF-α, are commonly secreted in psoriasis and atopic dermatitis-like skin conditions [6]. Calcineurin, a protein in the signaling pathway for T-cell activation, stimulates nuclear factors of activated T cells (NFAT) that are responsible for the transcription of inflammatory cytokines [7]. This cytokine secretion accelerates the differentiation of the T-cell subpopulation and the proliferation of keratinocytes. Hence, it causes disruption and irritation in the skin barrier, resulting in cellular alteration in inflammatory conditions like atopic dermatitis and psoriasis [6].

Potent immunosuppressants such as tacrolimus, a calcineurin inhibitor, disrupt the activation of T cells by binding to intracellular immunophilin proteins to block calcineurin from producing interleukins and other pro-inflammatory cytokines. The binding to intracellular immunophilin proteins forms a complex that results in reducing the transcription of T-cell growth factors such as interleukins (IL-2 and IL-4). Moreover, it also inhibits the release of inflammatory cytokines from mast cells and basophils, therefore making it effective in treating skin inflammation [8,9]. Corticosteroids and tacrolimus are recommended for the primary treatment of skin inflammation. However, topical corticosteroids are often associated with adverse effects on the skin.

In contrast, topical tacrolimus used in patients with atopic dermatitis is well-tolerated, effective, and enhances the quality of life [10]. However, due to its poor solubility, tacrolimus tissue penetration and skin bioavailability lead to reduced efficacy and treatment optimization challenges. The exhibition of low oral bioavailability, high interindividual and intraindividual variability, and poor absorption rates restrict its achievement of therapeutic effectiveness [11]. Tacrolimus undergoes first-pass metabolism in the stomach and liver, resulting in a bioavailability of approximately 21%. The variability in tacrolimus release profile among patients and lack of target specificity causes immunosuppression, affecting the entire immune system [12]. Due to its high lipophilicity, low solubility, and stability issues, tacrolimus exhibits a poor absorption profile. Therefore, it is necessary to develop a drug delivery system to optimize its stability with enhanced therapeutic profile.

Microemulsions as a drug delivery system have the potential to enhance the therapeutic profile of lipophilic drugs like tacrolimus by forming a stable formulation [13]. The capacity to solubilize both water-soluble and fat-soluble molecules in microemulsion makes them more efficient and promising. Notably, microemulsions are highly effective for precise drug delivery due to their small droplet size, large surface area, and thermodynamic stability [14,15]. The homogeneous mixture of two immiscible liquids (oil and water) stabilized by a surfactant results in the spontaneous formulation of an isotropic, transparent, and stable mixture [15]. The high percentage of surfactants in a microemulsion formulation acts as an emulsifier, causing it to self-assemble and form a micelle-like structure. The presence of a surfactant decreases the interfacial tension and results in a thermodynamically stable formulation [16,17]. It also enhances the membrane permeability profile by improving drug absorption [13]. Considering these attributes, this research focuses on developing microemulsion to serve as a delivery system for tacrolimus. Compared to traditional drug delivery strategies, nanomedicine-based drug delivery systems have the potential to surpass biological barriers and show improved therapeutic efficiency. However, moving from the development phase to clinical translation is associated with many trials, errors, investments, and risks. Controlling the critical properties of nanoemulsion formulations, such as their composition, particle size, charge, and other attributes, poses significant challenges during manufacturing. To account for this, the Quality by Design (QbD) approach is currently used to develop and optimize nanoemulsion formulation. The approach of QbD is outlined in ICH (International Conference on Harmonization) Q8, Q9, Q10, and Q11 guidelines [18,19,20,21]. These guidelines focus on the development of products based on a quality system, risk management, and a comprehensive understanding of the manufacturing process [22]. The application of QbD is explored in micro-molecules that are hydrophobic to investigate the impact of parameters on critical quality attributes (CQAs), including particle size, uniformity, zeta potential, drug release pattern etc. Following that, the application of the design of experiments (DoE) in QbD allows us to have a design space based on the main effects and interactions of the parameters involved. Thus, the roadmap of QbD directs the manufacturing of the product with a robust process while ensuring that the Quality Target Product Profile (QTPP) is achieved [23].

We implemented QbD to develop and optimize a stable tacrolimus-loaded microemulsion suitable for topical application in skin inflammation. The quality-driven systemic and risk-based strategy was utilized to develop the microemulsion from scratch. This approach began with the identification of QTPP with predefined CQAs. Following that, formulation and process screening were conducted for further exploration. The Failure, Mode, Effects, and Criticality Analysis (FMECA) was utilized to highlight the factors that were most likely to be critical for product quality and fully explore the design space [24]. Several earlier studies on QbD-driven nanoformulation-based development [24,25,26,27] utilized the D-Optimal design of experiments (DoE) that supported investigating multiple factors with continuous and discrete values with a minimal number of runs [26]. Moreover, the D-optimal criterion explains the effects and interactions of independent factors on the response factors within the design space. Statistical analysis using multiple linear regression (MLR) was used to determine the correlations between factors and CQAs.

The aim of the presented QbD strategy was to identify the critical parameters for manufacturing a stable microemulsion formulation. The optimized tacrolimus-loaded microemulsion with small particle sizes (30–50 nm) is intended for topical administration and treatment of skin inflammation. The implemented QbD approach allows us to statistically describe and correlate the critical variables. The presented microemulsion required optimization of formulation and process parameters to achieve QTPP [24]. The screening studies identified both composition and process parameters for further optimization. Pre-formulation studies revealed that a combination of low and high-energy emulsification methods was needed to form a stable microemulsion with the desired particle size (30–50 nm).

Once the process was established, the FMECA-based risk assessment identified formulation and process parameters that were potentially critical for product quality. After that, the application of the D-Optimal design of experiments enabled us to define a design space. Within the design space, the CQAs of the microemulsion candidates were significantly impacted by processing parameters in the microfluidizer. The DoE trials were assessed based on the CQA specifications. Out of eight microemulsion candidates, one formulation was selected for loading tacrolimus to meet CQA specifications. Furthermore, multiple linear regression (MLR) was performed to study the effects of parameters and their interactions.

The QbD framework in this study led to the development and optimization of a stable tacrolimus-loaded oil in water (o/w) microemulsion formulation, meeting the CQA specifications (particle size, dispersity index (DI), drug loading, serum, and filtration studies) with a robust manufacturing approach. It further highlights the success of the three-fold scalability of the microemulsion by utilizing the optimized manufacturing approach. The findings demonstrate an enhanced tacrolimus loading in the microemulsion with a slow and extended-release pattern, which could be a novel approach for localized delivery to treat skin inflammation. The slow-release profile of the microemulsion may serve to enhance patient compliance through reducing the frequency of topical application.

## 2. Materials and Methods

### 2.1. Materials

Tacrolimus-FK506 was purchased from Selleckchem, USA (Houston, TX, USA). Safflower oil was purchased from Sigma-Aldrich (St. Louis, MO, USA). Miglyol 812 was purchased from CREMER OLEO Product Division, Hamburg, Germany. Cremophor EL was purchased from Sigma-Aldrich (St. Louis, MO, USA). Transcutol (diethylene glycol monoethyl ether) was purchased from Sigma-Aldrich (St. Louis, MO, USA). PEG 400 was purchased from Sigma-Aldrich (St. Louis, MO, USA). 10× Phosphate Buffer solution was purchased from Quality Biological, Inc. (Gaithersburg, MD, USA). The syringe and 0.22 µm filter were purchased from EMD Millipore (Burlington, MA, USA) and used in filtration studies. Dulbecco’s Modified Eagle Medium (DMEM) from Gibco-BRL (Rockville, MD, USA) and 20% fetal bovine serum (FBS, ATCC 3020-20) were used in serum studies.

### 2.2. Methods

The pre-formulation and process screening were performed to select excipients and identify critical formulation and process parameters. The initial formulation screening was completed by changing the oil phase in the microemulsion (safflower oil or Miglyol 812N), the aqueous phase (Phosphate Buffer Saline (PBS) or water), adding a single or combination of surfactants (Cremphor EL or PEG 400), changing the magnetic stirring time (30 min or 60 min or 120 min), and utilizing different processing methods (Titration or Sonication or Microfluidization). Following that, a rigorous risk assessment was performed based on FMECA to identify potentially critical formulation and process parameters involved. Based on that, an 8-run D-Optimal DoE was utilized to select the microemulsion candidate. The trials included different percentages of surfactant and co-surfactant (high or low), titration rate (high or low), and processing pressure during microfluidization (high or low) as independent variables, keeping the oil percentage and stirring time constant.

#### 2.2.1. Production of Microemulsions: Application of Low Energy

In the pre-formulation screening, microemulsion was produced at 200 mL scale via titration method. Firstly, the oil (safflower oil or Miglyol 812N) and the surfactant (Cremophor EL alone or in combination with PEG 400) were pre-mixed for 15 min at 300 RPM by magnetic stirring in a tall-shaped beaker. After that, the co-surfactant (Transcutol) was added to the same beaker and continued with magnetic stirring for 15 min at 300 RPM. Secondly, the stirring rate was adjusted to 350 RPM, and the dropwise addition of 1× PBS (diluted PBS from 10× PBS stock solution) was performed at a specified rate of 4 mL/min. The RPM and mixing time were set based on a previous study on microemulsion [24]. After completing the titration, the microemulsion continued to be stirred for (30 min or 60 min or 120 min). All microemulsions were prepared at room temperature. In Section 3.2 (below), a detailed recipe has been given for each trial prepared using a low-energy method. 

#### 2.2.2. High Energy Processing of Microemulsions: Sonication and Microfluidization

After manufacturing the microemulsion by titration method following the same procedure described in Section 2.2.1, it was processed with two different types of high-energy processes, including sonication and microfluidization. For sonication, Sonic Dismembrator (Model 500, Fisher Scientific, Hampton, NH, USA) was used, and microemulsion was processed with 5 pulses with 29% amplitude. For microfluidization, LM20 Microfluidizer^®^ (Microfluidics Corporation, Westwood, MA, USA) was used to process with 6 passes at different pressures (15,000 to 20,000 psi) in the microfluidizer.

#### 2.2.3. Optimized Manufacturing Process Applied in DoE

Microemulsion was produced at 200 mL scale via titration method and then processed via microfluidizer. At first, the oil phase (safflower oil) and surfactant (Cremophor EL) were pre-mixed for 15 min at 300 RPM by magnetic stirring in a tall beaker. The co-surfactant (Transcutol) was added to the same beaker and continued mixing for 15 min at 300 RPM by magnetic stirring. Then, the stirring rate was adjusted to 350 RPM, and 1× PBS (diluted PBS from 10× PBS stock solution) was added dropwise to the beaker. For titration, the 1× PBS addition was performed at a specified rate (4 mL/min or 12 mL/min). After completing the titration, the microemulsion continued to be stirred for 2 h. All microemulsions were prepared at room temperature. After stirring, the microemulsion was poured into the inlet reservoir of the LM20 microfluidizer^®^ and processed with consecutive 5 passes at the specified pressure of the microfluidizer (15,000 psi or 20,000 psi). The interaction chamber of the LM20 microfluidizer^®^ was kept on ice throughout this process to maintain the processing temperature from 15 °C to 20 °C. After selecting the microemulsion candidate based on CQAs, the microemulsion was scaled up to 600 mL (3-fold) following the optimized manufacturing process.

#### 2.2.4. Tacrolimus-Loaded Microemulsion

The tacrolimus-loaded microemulsion was manufactured at a 200 mL scale. In addition to the same manufacturing process, the overnight mixing of 360 mg of tacrolimus with 6.1% of transcutol was also performed. Following that, the optimized formulation and process parameters, including 6% safflower oil and 22.5% Cremophor EL, were mixed at 300 RPM for 15 min. Then, the mixture of tacrolimus and transcutol was added and continued stirring for 15 more minutes. The stirring rate was adjusted to 350 RPM, and 1× PBS titration was performed at a 4 mL/minute rate following magnetic stirring for two hours. Then, the microemulsion was processed via LM20 microfluidizer^®^ with five passes at 20,000 psi processing pressure.

### 2.3. CQA Specification Studies

#### 2.3.1. Droplet Size and Size Distribution Measurements

After manufacturing the microemulsions, the dilution factor for measuring the particle size was optimized. All microemulsion samples were analyzed for droplet size and size distribution upon dilution 1:80 (*v*/*v*) in de-ionized water. The particle size, DI, and particle size distribution were measured using dynamic light scattering (DLS) measurement (Zeta-sizer NanoZS, Malvern, UK). The measurement was conducted at the temperature of 25 °C using a light scattering angle of 173°.

#### 2.3.2. Filtration Study

The filtration test was performed by filtering 10 mL of microemulsion through a 0.22 μm pore size Millex-GS syringe filter and was stored at room temperature. The filtered microemulsion was diluted 1:80 *v*/*v* in de-ionized water. The droplet diameter and DI of the filtered and unfiltered microemulsions were measured by dynamic light scattering (DLS) measurement on Zeta-sizer NanoZS (Malvern, UK).

#### 2.3.3. Serum Study

The serum stability of microemulsions was studied to assess their behavior in biological environments at 0 h and 72 h by incubating at 37 °C. To perform this study, the microemulsion was diluted at 1:80 (*v*/*v*) in 20% fetal bovine serum (FBS) in Dulbecco’s Modified Eagle’s Medium (DMEM). The particle size was measured at both 0 h and 72 h using dynamic light scattering (DLS).

### 2.4. Design of Experiments

Two-level, four-factor D-Optimal DoE was developed using JMP^®^ (version 17.2.0, SAS Institute Inc., Cary, NC, USA) software. The percentage of oil (6%), stirring time (2 h), and number of passes during microfluidization processing (5) were determined based on screening experiment and prior literature and our laboratory experience. The constraints defined as variables in the design space were the percentage of surfactant (22.5%, 27.5%), percentage of co-surfactant (7.5%, 6.1%), titration rate (4 mL/min, 12 mL/min), and pressure in microfluidization (15,000, 20,000 psi). For final process optimization an 8-run-based D-optimal design of this experiments was implemented using JMP^®^ software to evaluate the impact of variables.

### 2.5. Statistical Analysis

Multiple linear regression (MLR) was performed to define the relation between independent variables and response variables. The main effects of the independent variables were analyzed. The fit model analysis was performed in JMP^®^ software, which demonstrated the *p*-value involved in individual effects, and based on that, the impact of each variable was evaluated.

### 2.6. Drug Quantification

The drug quantification was performed using the Reverse-Phased High-Performance Liquid Chromatography (RP-HPLC) technique on Dionex Ultimate 3000 UHPLC (Thermo Fisher Scientific, Waltham, MA, USA). An optimized Reverse Phase-HPLC (RP-HPLC) instrument method from prior publication [28] was utilized for quantifying TAC-loaded microemulsion maintaining the flow rate at 0.75 mL/min, column temperature 60 °C and post-column 10 °C, UV channel setting at 210 nm. The mobile phase containing acetonitrile (70):Water (30):Phosphoric Acid (0.05) was utilized on column C18 (Phenomex column) specific to tacrolimus. The standard samples for tacrolimus were prepared from 1 to 20 µg/mL concentration for the calibration curve. For quantification, the TAC-microemulsion (1.8 mg/mL) was diluted into HPLC-grade acetonitrile to a final concentration of 500 µg/mL and then further diluted into the mobile phase to make 20 µg/mL. The samples were prepared in triplicates and analyzed based on the area under the curve (AUC) observed in the chromatogram.

### 2.7. In Vitro Analysis of TAC Release from TAC-ME

The release profile of tacrolimus from microemulsion was analyzed by performing in vitro analysis using 3.5 MWCO (molecular weight cut-off, CAT#68035, Thermo Fischer Scientific, Waltham, MA, USA). The dialysis bag was kept at 37 °C to mimic human body temperature, and the receptor compartment was PBS and 0.1% tween 80 (pH 7.4) in a total of 15 mL as release media [28]. An amount of 1 mL of TAC-ME and the equivalent amount of Free-TAC solution (TAC in acetonitrile) were placed in the dialysis bag. The dialysis bag was switched into fresh release media at regular intervals (1, 4, 6, 24, 48, 72, 120, 168 h), and quantification was performed using the same RP-HPLC method. The cumulative amount and percentage of drug release versus time were plotted to evaluate the drug release profile from TAC-ME and Free-TAC solution.

## 3. Results

**Figure 1:**
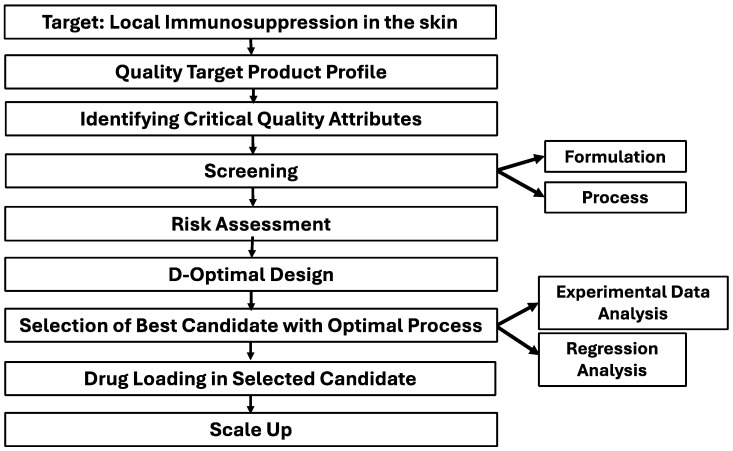
Flow chart of step-by-step QbD approach adopted in this study for achieving the QTPP.

For the development of tacrolimus-loaded microemulsion, a structured method of development with predefined goals was followed, which focused on having a comprehensive understanding of the process and quality of the product. This approach was built on scientific concepts and quality risk management to guarantee robust process control and product quality. The step-by-step approach (Figure 1) is defined as the QbD approach.

The objective of applying the QbD strategy was to develop a microemulsion formulation for loading tacrolimus, which can be used to suppress the immune response to skin inflammation. The QTPP was determined to formulate a stable, transparent oil-in-water microemulsion formulation for topical application. Considering the characteristics of an oil-in-water microemulsion with a smaller particle size, it was preferred for application to the skin. The particle size of the microemulsion was targeted between 30 nm and 50 nm with a distribution index of less than 0.3 [24]. The range of particle sizes was set to ensure efficient tissue penetration to the target site on the skin because microemulsions with a smaller particle size have a high surface area and lower interfacial tension that can improve the solubility and bioavailability profile [29]. More than 50% tacrolimus loading in microemulsion was targeted to achieve a slow and extended-release profile for treating skin inflammation in a controlled manner.

### 3.1. Critical Quality Attributes (CQAs) Identification

The CQAs were identified for the target product, which must fall within specific ranges to ensure product quality (Table 1). The Day 1 microemulsion diameter or particle size was selected to 30–50 nm because smaller particle size with large surface area facilitated better permeation. The lower surface tension and concentration gradient in microemulsion drive the drug permeation in the skin when applied topically [30]. DI ≤ 0.3 was targeted for maintaining uniform distribution of particle size and improving efficacy in drug delivery, as inconsistency in the distribution of particle size might lead to uncontrolled drug release, impacting the quality of the product. Moreover, it was targeted to achieve less than 10% deviation in particle size and ≤0.3 DI after filtering the microemulsion to evaluate its behavior under stress, as the product had to withstand stress during long-term storage and transportation for maintaining the quality attributes. Similarly, <10% deviation in particle size and ≤0.3 DI was targeted to achieve after serum studies. The serum study is performed to assess the behavior of microemulsion when interacting with the biological environment [24]. The drug loading capacity of the microemulsion was targeted to achieve more than 50%, as effective drug loading in the carrier was important for enhancing bioavailability and ensuring therapeutic effectiveness.

### 3.2. Screening: Pre-Formulation and Process

**Figure 2:**
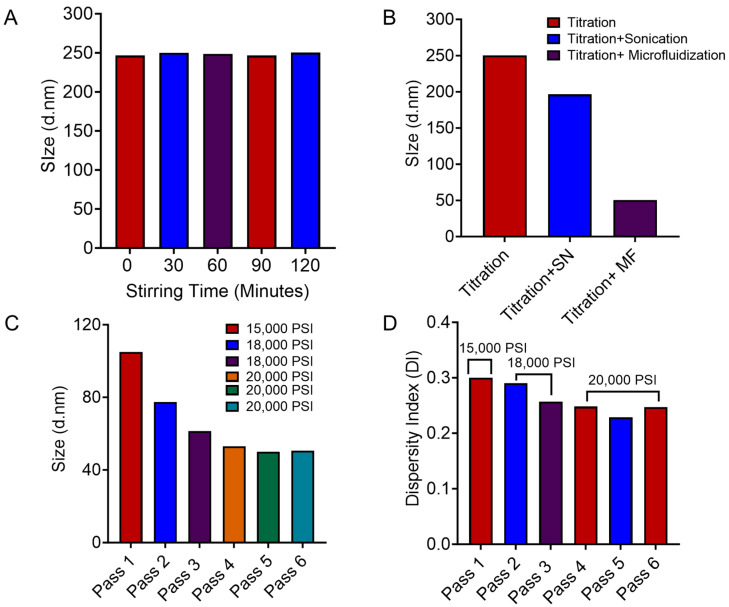
The data during the processing screening are presented in the graphs (**A**–**D**). (**A**) Denotes the particle size of microemulsion from (Trial A) at each point during magnetic stirring. (**B**) Demonstrates a comparison of particle size after the application of low energy (Titration) and in combination of low and high energy (Titration with Sonication/Microfluidization). (**C**,**D**) Demonstrates the reduction in particle size and DI with an increase in microfluidization pressure (15,000 to 20,000 psi).

After the identification of CQAs, pre-formulation (Table 2) and process screening (Figure 2) were performed to select excipients and identify critical formulation and process parameters. During the screening phase, oil-in-water-based microemulsion trials were developed using both low-energy and a combination of low and high-energy methods.

#### 3.2.1. Pre-Formulation Screening

The initial formulation screening was performed by changing the oil phase in the microemulsion (safflower oil or Miglyol 812N), the aqueous phase (PBS or water), adding a single or combination of surfactants (Cremphor EL or PEG 400). Following that, the impact of different types and percentages of oil phases, surfactants, and co-surfactants, as well as the choice of aqueous phase, was studied through different microemulsion trials. As a result, changing the type and percentage of formulation components showed a significant impact on the particle size and DI of microemulsions. The fatty acid chain length in different types of oil, their required HLB for surfactants for emulsification, and the oil-to-surfactant ratio are critical factors to be considered in microemulsion formation. These factors influence the interfacial surface tension to be lower or higher, impacting the particle size and overall stability of the microemulsion [31]. In Table 2, safflower oil-based microemulsion was prepared using Cremophor EL as a surfactant, and Transcutol as a co-surfactant in PBS, and particle size was >250 nm with a DI of 0.217, which was out of specification according to the QTPP (Trial A). Long chain-based safflower oil impacted the particle size to be greater than 250 nm. To explore further, a combination of surfactants (Cremophor EL: PEG 400) was added to the same formulation in a 1:1 ratio (Trial B). However, the particle size was >700 nm; the formulation was unstable and eventually became phase-separated. In this trial, the ratio of safflower oil and Cremophor EL decreased from 1:4.5 to 1:2.2 ratio, which significantly impacted the particle size. The 1:1 ratio of Cremophor EL: PEG 400 was not optimal for stabilizing the interface of microemulsion and formed larger particles, later resulting in phase separation. Subsequently, PEG 400 was removed (Trial C), and keeping the same formulation, the percentages of components were decreased, which also resulted in particle size out of specification (339 nm) and an unstable microemulsion formulation. The result from Trial C indicates that the 13.25% concentration of the surfactant (Cremophor EL) was not enough to reach critical micelle concentration (CMC) and stabilize the system. Although the oil-to-surfactant ratio was maintained at 1:4.5, the low percentage of the components was not optimal to stabilize the formulation. In the next trial, the oil phase was changed to a medium chain oil phase as Miglyol, keeping the Cremphor EL. 

Transcutol was used as a surfactant and co-surfactant, respectively, and PBS was used as the aqueous phase. The trial resulted in a particle size of less than 20 nm and a DI of 0.043. However, during the bi-continuous phase of preparing this trial, gel-like consistency was observed, indicating that the components might not have achieved complete homogeneity. Significant differences in homogeneity and particle sizes were observed in trials consisting of different oil phases (safflower Oil and Miglyol). By considering the stability of microemulsion trials, formulation with safflower oil (6%) was utilized to investigate further the impact of aqueous phase change on the particle size and DI. To investigate the impact of the aqueous phase, instead of PBS, water was utilized in another trial. However, no significant difference was observed in changing the aqueous phase. Throughout these trials (Table 2), it was observed that the choice and percentage of components in the formulation, specifically oil and surfactant, have a significant impact on the particle size and DI of microemulsion. After pre-formulation screening, the percentage of oil was fixed to 6% in microemulsion for further trials.

#### 3.2.2. Process Screening

The pre-formulation screening was performed based on changing the formulation parameters and manufactured using a low-energy method (titration). The microemulsions were spontaneously formulated after titration, followed by magnetic stirring. The magnetic stirring time after titration was fixed to 120 min for better homogeneity. However, no impact on particle size was observed for changing stirring time (Figure 2A). Among the screening trials, only two microemulsions formulated with safflower oil, Cremphor EL, and Transcutol, in higher percentages, were observed to be stable in both PBS and water. However, the particle size was higher than the targeted profile (30–50 nm). To investigate the screening process further, a combination of low and high-energy methods was applied to compare the impact on the particle size (Figure 2B). At first, sonication as a high-energy method was applied along with titration for processing the microemulsion, and the particle size of the microemulsion decreased to 200 nm with five pulses. Furthermore, microfluidization was also utilized as a high-energy method, along with titration, for processing microemulsion. It was observed that using the microfluidizer and titration method effectively with six passes reduced the particle size below 50 nm (Figure 2B,C), meeting the range to achieve the QTPP. Figure 2C,D shows that the increase in pressure during microfluidization in each pass resulted in a reduction in the particle size and DI. However, the microemulsion was fixed to pass through the microfluidizer five times based on the uniform distribution of particle size (Supplementary Figure S1). The combination of titration and microfluidization was adopted for processing microemulsion, as this approach successfully reduced the particle size within the desired range according to QTPP. However, using the combination of titration and sonication approach was not effective enough to reduce the particle size within the desired range. Therefore, the combined approach of titration and microfluidization was selected to apply in the DoE trials for manufacturing the microemulsion formulation.

### 3.3. Risk Assessment

The risk assessment can be defined as a systemic approach that assists in identifying, analyzing, and prioritizing critical factors that can have a significant impact on product quality [24,32]. The quality of the final product is controlled by various parameters, including manufacturing conditions, processing parameters, material attributes, etc. There are multiple variables involved in each unit operation, starting from dispensing to transferring the final product to the main storage container. To maintain the quality of the final product, the critical variables need to be identified. For that, an FMECA-based risk assessment strategy was utilized in this study to systemically rank the most critical variables from different unit operations [24]. This strategy works as a guide to construct the DoE based on quantitative evaluation of identified risk factors. The risk factors involved in the manufacturing approach were assigned to risk priority numbers (RPN) based on the frequency of occurrence (O), severity (S), and detectability (D).

RPN = Severity (S) × Frequency of Occurrence (O) × Detectability (D)

In this study, low and high-energy methods (titration and microfluidization, respectively) were combined to manufacture microemulsion. The microemulsion was first formulated with the titration method and then processed in the microfluidizer to achieve the desired particle size. The two primary unit operations of this manufacturing process involved titration and microfluidization. These two-unit operations have many variables to be considered, such as PBS addition rate, stirring time, stirring rate, temperature, vessel geometry, magnetic bar dimension, pressure of microfluidizer, number of passes, interaction chamber, etc. Moreover, the formulation parameters are known to be the most critical in microemulsions [24]. For that, the risk factors involved in both processing and formulation parameters were ranked by using RPN values.

During microemulsion production in the screening phase, risk factors involved in formulation and processing were identified. Subsequently, these risk factors were analyzed and ranked by calculating the risk priority number (RPN), considering three factors, including severity, frequency of occurrence, and detectability. Thus, an RPN value was assigned for each risk factor potential, leading to failure in achieving CQAs. For each of these factors, a measurement scale was selected starting from low to high (1 to 5), as shown in Table 3, which helped to rate each risk factor based on RPN value. By multiplying different scales of severity, frequency of occurrence, and detectability, the 3 RPN range was decided to classify all the risk factors. Therefore, the RPN value for high-risk factors was 75–125; for medium risk, the RPN value was 50–74, and for low risk, the RPN was <50. All risk factors were categorized based on this RPN range.

**Figure 3:**
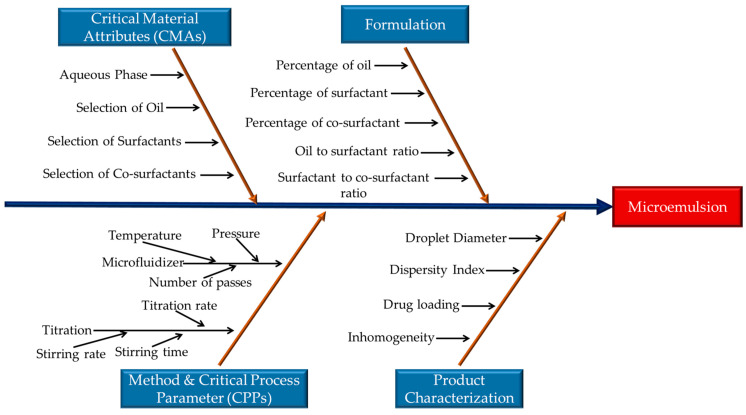
Ishikawa fishbone diagram representing potential causes contributing to the failure to meet the CQAs.

Formulation parameters, especially oil-to-surfactant ratio, amount of surfactant and co-surfactant, and oil percentage, significantly impact CQAs [24]. Furthermore, the titration rate and stirring time might have an impact on microemulsion droplet size as well. Moreover, processing microemulsion in a microfluidizer is not a typical approach. It was considered that the number of passes, temperature, and pressure of the microfluidizer might have a significant impact on CQAs based on theoretical knowledge and prior trials. The critical factors identified as failure modes are illustrated in Figure 3. The deviation in ranges of these factors may result in failure in producing a microemulsion that meets specified CQAs.

Table 4 summarizes the classification of all identified risk factors (process and formulation) into three categories (low, medium, and high) based on the different levels of RPN values from Table 3. Based on the RPN values, processing parameters, including stirring time, stirring rate, and temperature, were considered to have a low-risk profile (RPN < 50) for failing to meet the particle size, DI and drug loading, and a medium-risk profile (RPN = 50–74) for failing to achieve homogeneity. The percentage of oil, surfactants, co-surfactants, PBS addition rate, and pressure in the microfluidizer were considered to have a high-risk profile (RPN = 75–120), contributing to failure in meeting CQAs. During the screening trials, all risk factors were prioritized, and the identified high-risk factors that were selected are listed in Table 5. The process and formulation parameter with the highest RPN value (75–120) was recognized as the high-risk factor.

In Table 5, high-risk factors were identified, which included titration rate (too low or too high), percentage of oil (too low or too high), percentage of surfactant, co-surfactant (too low or too high), and processing pressure in microfluidization (too low or too high). The imbalance in percentage in oil, surfactant, and co-surfactant may lead to aggregation, coalescence, or even phase separation, impacting the CQAs, including droplet diameter, DI, drug loading, homogeneity, etc. Moreover, extremely high or low pressure during the processing of microemulsion in a microfluidizer may potentially involve failure to meet CQAs. Therefore, the identified high-risk factors were utilized to apply in the DoE to explore the relationship between the high-risk factors and response factors. This FMECA approach has been utilized in some other studies as an assessment tool for the successful identification and management of critical risks [24,33].

### 3.4. Design of Experiments: D-Optimal Design

**Figure 4:**
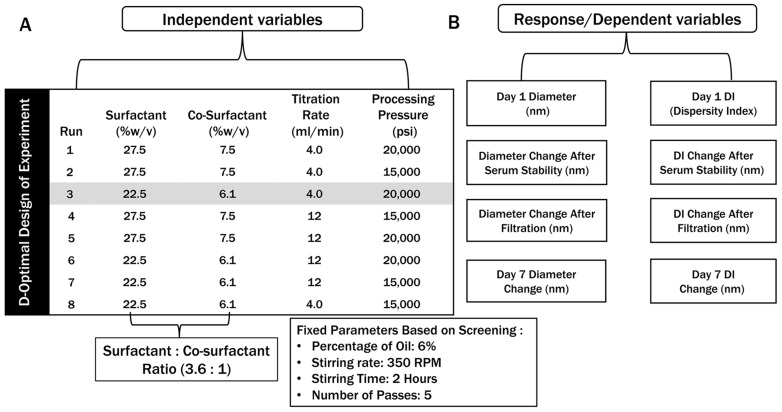
(**A**) Runs obtained from applying a 2-level D-optimal design of this experiment; the parameters used as variables in the D-optimal design are independent. (**B**) The response/dependent variables considered to measure for each run in this experiment.

Based on the initial screening of formulation and process, the selected oil (safflower oil) and percentage of oil (6%), stirring rate (350 RPM), stirring Time (2 h), and number of passes (five) were fixed. Following the FMECA, eight runs based on two-level, four-factor D-Optimal DoE were developed for studying the critical formulation parameters and critical process parameters, including the percentage of surfactants and co-surfactants, titration rate, and processing pressure of microfluidizer (Figure 4A). The parameters were considered as independent variables in this experiment. The response variables presented in Figure 4B were considered as dependent variables.

The CQAs, as response variables, were measured for each run, and results were analyzed based on the specifications from the QTPP (Table 6). Runs that were not able to meet CQA specifications, including particle size and DI of Day 1 and Day 7, DI ≤ 0.3, 10% change after filtration, and serum stability studies, were excluded. Among eight runs, only one candidate (**Run 3**) met all the specifications of the targeted profile. The selected microemulsion was stable and transparent, and the droplet diameter was <50 nm on Day 1 and Day 7. The DI was below 0.3. In the filtration and serum studies, the particle size did not deviate by more than 10%, and the DI was below 0.3, indicating that it met all the CQA specifications. The parameters in the selected run were further utilized for a three-fold scale-up and drug loading. Moreover, it was observed that the desired particle size on Day 1 was achieved from runs 1, 5, and 6. However, they did not meet CQA specifications during stress and serum studies and were excluded from further analysis. Only one of them met all the CQA specifications. However, all the microemulsion trials were stable and maintained the particle size and DI in a constant manner in this design space.

### 3.5. Assessment of DoE Trials

**Figure 5:**
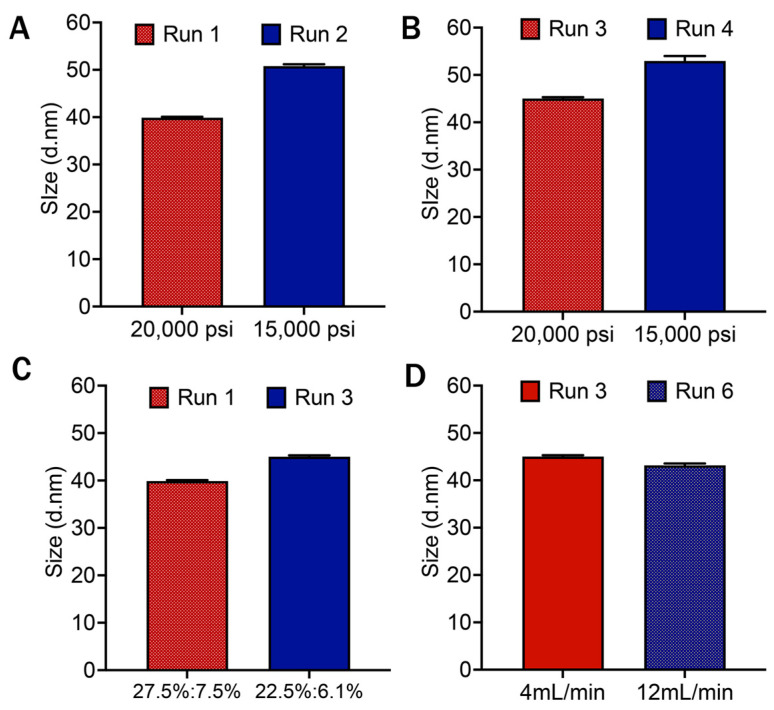
Experimental analysis from the D-Optimized DoE trials. (**A**) Illustrates the comparison of particle size of Day 1 for runs 1 and 2, where the percentage of surfactant and co-surfactant was higher (27.5%, 7.5%), and titration rates were 4 mL/min and 12 mL/min for both runs, but processing pressures were 20,000 and 15,000 psi, respectively. (**B**) The comparison of particle size with different processing pressures (20,000 and 15,000 psi) where percentages of surfactant and co-surfactant (22.5%, 6.1%) were low, with titration rate (4 mL/min) for both run 3 and run 4. (**C**) Represents the impact of high and low percentages of surfactant and co-surfactant combination in particle size on Day 1 (constant parameters for both run 1 and run 3: 20,000 psi, 4 mL/min). (**D**) Shows the effect of low (4 mL/min) and high (12 mL/min) titration rates in particle size on Day 1 for run 3 and run 6 (constant parameters for both run 3 and run 6: 20,000 psi, low percentages of surfactant and co-surfactant).

From the experimental data analysis, it was observed that microemulsions that were manufactured at high pressure of microfluidizer (20,000 psi) regardless of a surfactant or co-surfactant amount and titration rate, met the specified range for particle size in both Day 1 and Day 7, indicating that the pressure of microfluidizer had a significant impact on the particle size of the microemulsion. Figure 5A demonstrates Day 1 particle size from run 1 and run 2, where both runs maintained constant parameters (higher surfactant and co-surfactant, low titration rate) except the pressure of microfluidizer (20,000 or 15,000 psi). Our results indicated that changing the processing pressure in microfluidization substantially impacted the particle size (*p*-value < 0.00005) in trials with higher surfactant concentrations. Similarly, Figure 5B indicates that changing the processing pressure in the microfluidizer (20,000 or 15,000 psi) has a significant effect (*p*-value < 0.005) on particle size for run 3 and run 8 as well. Both run 3 and run 8 maintained consistent parameters (lower surfactant and co-surfactant, low titration rate) and different processing pressures in the microfluidizer. Figure 5A,B indicates that, regardless of the high and low percentage of surfactant and co-surfactant, the processing pressure in a microfluidizer had a significant impact on the particle size of the product. Another comparative analysis demonstrated that (Figure 5C) changing the percentage of surfactant and the co-surfactant combination also impacted the particle size of the microemulsion (*p*-value < 0.00005). Run 1 (high percentage of surfactant and co-surfactant) and run 3 (low percentage of surfactant and co-surfactant) resulted in particle sizes of 39.88 nm and 45.02 nm, respectively (run 3 > run 1). However, both runs were in the specified range of particle size (<50 nm). Moreover, Figure 5D shows the impact of changing the titration rate (4 mL/min or 12 mL/min) alone while keeping other parameters constant. The change deviated the particle size diameter from 45.02 nm to 43.15 nm (*p*-value < 0.005). However, the level of significance is lower for titration rate compared to other parameters, and the particle size observed for both runs was within the range of <50 nm. 

### 3.6. Multiple Linear Regression (MLR) Analysis

**Figure 6:**
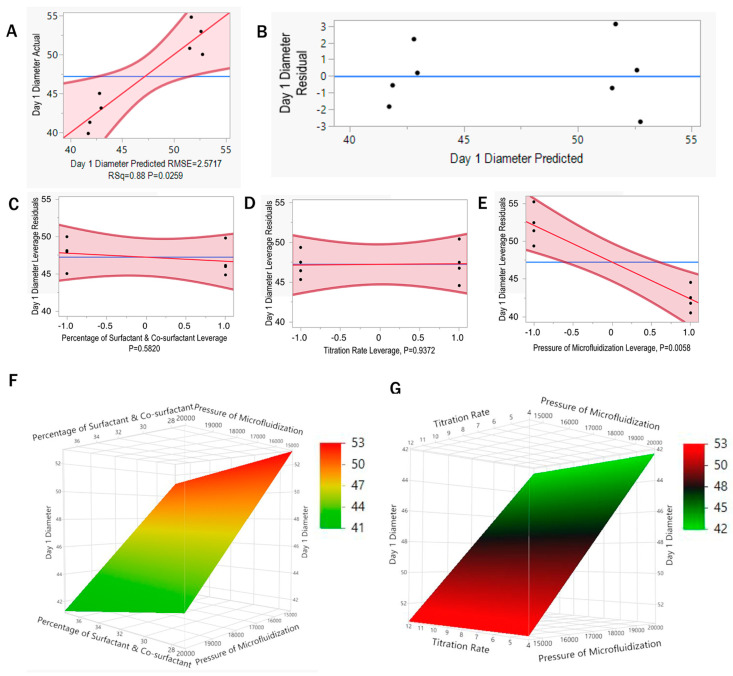
(**A**) Illustrates the actual vs. predicted value of the model showing a *p*-value < 0.05 (**B**) The residual vs. predicted plot of this model. (**C**–**E**) The plots demonstrate residual vs. leverage to describe the relationship between individual variables and particle size as response factors in Day 1 particle size diameter in microemulsion trials. The residual vs. leverage plot represents the individual main effects of independent variables. (**C**) Percentage of surfactant and co-surfactant, (**D)** titration rate, (**E**) pressure of microfluidization) for Day 1. (**F**,**G**) depict the response surface plots showing the relationship between independent variables percentages of surfactant and co-surfactant, titration rate, and pressure of microfluidization.

Multiple linear regression (MLR) was performed to show the relationship between individual and response variables involved in the design space of D-Optimal DoE. From the regression model analysis, the actual vs. predicted value of the model (Figure 6A), the residual vs. predicted model (Figure 6B), and the individual effects of independent variables are plotted (Figure 6C–E). The actual vs. predicted plot for Day 1 diameter (2A) indicated a *p*-value of 0.0259 (<0.05), suggesting that the parameters involved in this model were statistically significant for predicting Day 1 diameter of microemulsion. This model showed an R^2^ value of 0.88 and an RMSE (root mean standard error) value of 2.5717, suggesting that 88% of the variance could be explained by this model with a lower standard error value. In Figure 6B, the residual vs. predicted plot shows that variables are randomly scattered without showing any pattern, indicating an independent behavior of the variables. The residual vs. leverage plots in Figure 6C–E) demonstrated the impact of independent variables, including the percentage of surfactants and co-surfactants, the titration rate, and the processing pressure in the microfluidizer on Day 1 particle size of the microemulsion trials. In Figure 6C,D, it was observed that the mean of the model and mean of the samples intersected linearly and had a *p*-value of 0.5820 and 0.9372, respectively. The *p*-value indicates that both variables (percentages of surfactant and co-surfactant and titration rate) do not significantly impact the particle size diameter of the microemulsion. However, in Figure 6E, the meanings of the model and sample intersect diagonally and have a *p*-value of 0.0058, which is <0.05, indicating a statistically significant impact on the particle size of the microemulsion. The regression model and experimental analysis showed that the pressure used in the microfluidizer had the most significant effect on the particle size diameter of the microemulsion. Similar results were observed for the response variable Day 7 particle size diameter, where the *p*-value was 0.0094, which was <0.05, indicating a significant impact on the response variable. However, the percentages of surfactant and co-surfactant and titration rate had no substantial impact on particle size for Day 7 in this design space (Supplementary Figure S2). The response surface plots in Figure 6F,G depict the interaction of independent variables and a response variable. Figure 6F shows increased percentages of surfactant and co-surfactant, and pressure in microfluidization reduces the particle size within the desired range <50 nm. Additionally, Figure 6G showed the region where the decrease in titration rate and increase in pressure of microfluidization reduced the particle size <50 nm. The region where the particle size is falling out of the specified range >50 nm in Figure 6F,G can be considered as the edge of failure in meeting the CQA specification.

**Figure 7:**
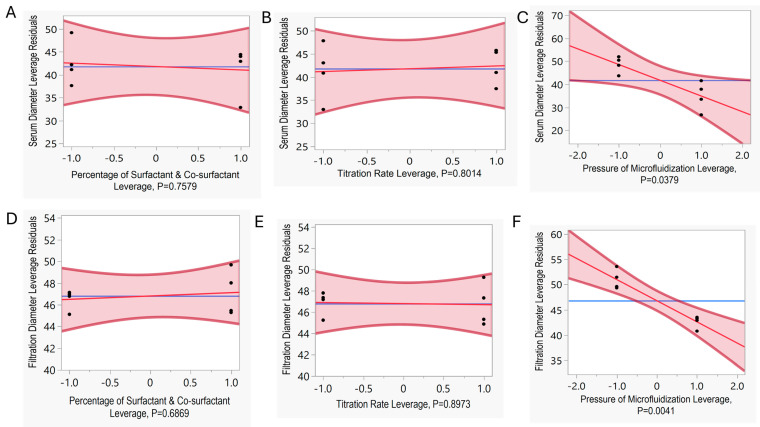
The plots demonstrate residual vs. leverage to describe the relationship between individual variables and particle size as response factors in particle size in serum stability study (**A**–**C**) and filtration study (**D**–**F**) in microemulsion trials. The residual vs. leverage plot represents the individual main effects of independent variables for particle size in serum stability study in (**A**) percentage of surfactant and co-surfactant, (**B**) titration rate, (**C**) processing pressure in microfluidization, and filtration study in (**D**) percentage of surfactant and co-surfactant, (**E**) titration rate, (**F**) processing pressure in microfluidization. The *p*-value < 0.05 indicates significant impact in the response variable.

Moreover, individual effects on particle size after serum and filtration study were analyzed as well. Figure 7A–C demonstrates the effect of individual parameters on serum diameter as the response variable. In Figure 7A,B, the mean of the model and mean of the sample intersect linearly, and the *p*-value is >0.05, indicating that both the percentages of surfactant and co-surfactant and titration rate have no significant impact on the particle size after serum study. Similar results were observed for particle size after the filtration study in Figure 7D,E); the *p*-value for the percentages of surfactant and co-surfactant and titration rate was >0.05. However, in Figure 7C,F, the plots demonstrate that the mean of the model and mean of the sample intersect diagonally, indicating that the processing pressure in the microfluidizer had a significant impact on the particle size after serum and filtration study. The *p*-values for the individual effect of processing pressure in microfluidization in particle size after serum and filtration were 0.0379 and 0.0041, respectively (Figure 7C,F), indicating a substantial impact on the particle size after serum and filtration study. However, only 70% of the variance in diameter after serum studies can be explained by this model, suggesting that there may be other variables involved in predicting diameter after serum study (Supplementary Figure S3A). Based on the R^2^ and *p*-value for predicting diameter after filtration, 90% of the variance can be explained by this model with a *p*-value of < 0.05, suggesting statistical evidence of the parameters involved in this model for predicting diameter after filtration study (Supplementary Figure S3C).

### 3.7. Selected Microemulsion Formulation

**Figure 8:**
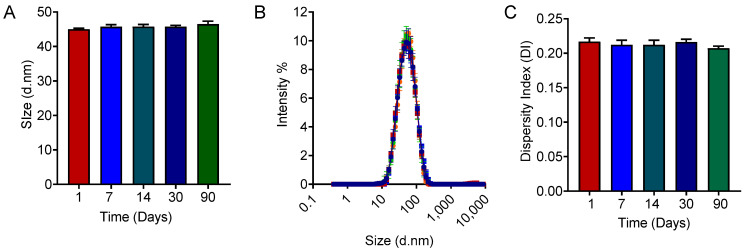
The assessment of the stability profile of selected microemulsion candidate: (**A**) represents the particle size, (**B**) the distribution of particle size, and (**C**) the DI of the selected microemulsion trial (Run 3) over a longer period. Data represents the average ± SD (n = 3).

Based on the results and summary of CQA studies, the selected candidate (run 3) out of eight runs successfully met the CQA standards, considering the QTPP. The independent variables for that run consisted of the low percentages of surfactants (22.5%) and co-surfactants (6.1%), low titration rate (4 mL/min), and high processing pressure (20,000 psi) in the microfluidizer (Figure 4A). The particle size and DI were assessed and resulted in 45.02 nm with 0.22 DI, which falls under CQA specification. Also, this candidate showed a uniform distribution of particle size, maintaining consistency over time. To monitor the consistency in the stability profile of this candidate, a long-term assessment was performed. The results showed that the microemulsion candidate maintained consistency in the particle size, DI (Figure 8A,C), along with a uniform distribution of particle size (Figure 8B) over time while it was stored at room temperature. As a result, it ensured that the selected microemulsion formulation could maintain a stability profile over a longer period, confirming a longer shelf life [34].

### 3.8. Scale Up

**Figure 9:**
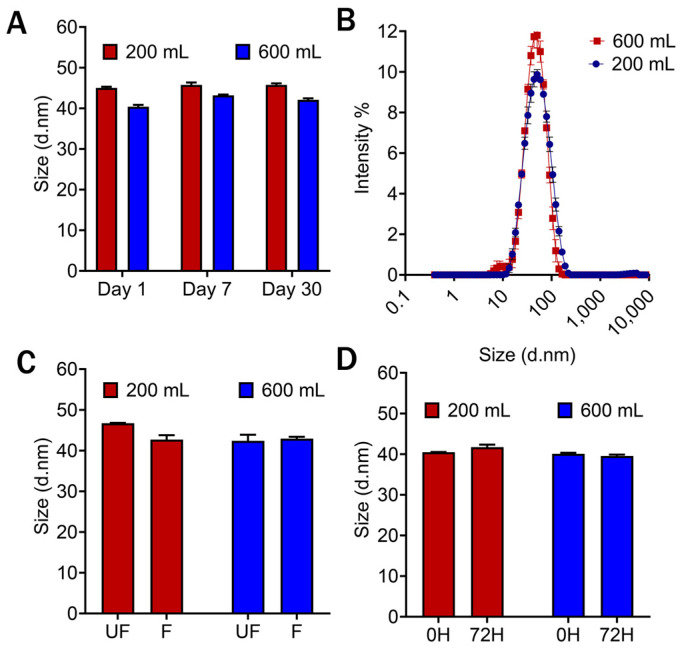
The comparison of 200 mL and 600 mL of drug-free microemulsion batches, where (**A**,**B**) represents the particle size and distribution of particle size for both microemulsions. (**C**) The 0.22 µ filtration study was conducted for both microemulsions (200 mL, 600 mL), and the particle size of unfiltered (UF) and filtered (F) microemulsions was measured. (**D**) Serum stability study was performed in 20% FBS in DMEM by incubating 37 °C, and particle size was measured for 0 H and 72 H. Data represents the average ± SD (n = 3).

From the D-Optimal design space, the optimized formulation and process were utilized for a three-fold scale up from 200 to 600 mL. Utilizing the optimized approach, the microemulsion was successfully scaled up, and it was observed that the scaled-up microemulsion also followed the same result as run 3. Therefore, the manufacturing approach for small-scale microemulsion was reproducible on a larger scale [24,35] and comparable to the 200 mL. The three-fold scaled-up microemulsion met the CQA specifications similarly to the small-scale microemulsion. A comparison between the small-scale microemulsion (200 mL) and large-scale microemulsion (600 mL) is illustrated in Figure 9. Both microemulsions met the desired particle size range (30–50 nm) with uniform particle size distribution (Figure 9A,B). In serum and filtration studies, no more than 10% change in particle size was observed for both 200 mL scale and 600 mL microemulsion (Figure 9C,D). This confirmed that the manufacturing approach was robust and could be utilized to scale up while maintaining the CQAs.

### 3.9. Drug-Loaded and Drug-Free Microemulsion

**Figure 10:**
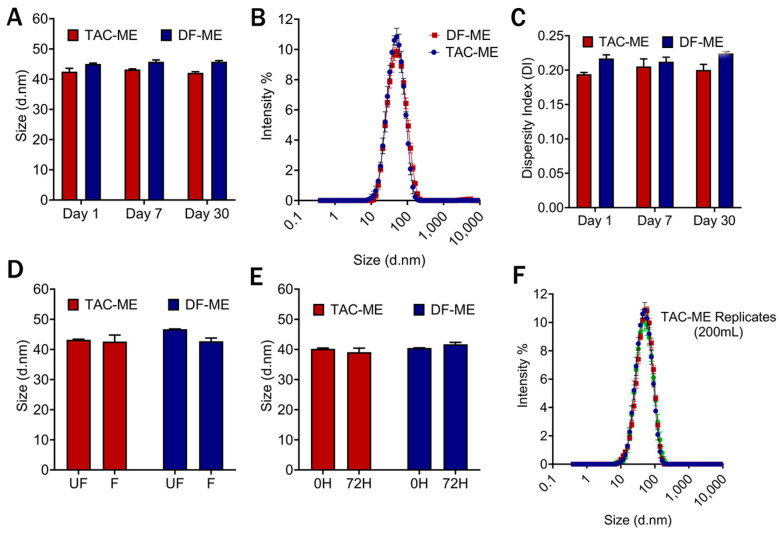
Characterization of drug-free microemulsion (DF-ME) and tacrolimus-loaded microemulsion (TAC-ME). (**A**) Overlay of one-month particle size assessment of DF-ME and TAC-ME, (**B**,**C**) The comparison of particle size distribution and DI of DF-ME and TAC-ME. (**D**) Impact of filtration in DF-ME and TAC-ME. (**E**) Impact on particle size after storing for 72 h in 20% FBS in DMEM containing culture media at 37 °C. (**F**) The particle size distribution of three replicate batches of tacrolimus-loaded microemulsion. Data represents the average ± SD (n = 3).

Following that, tacrolimus drug (1800 μg/mL) was loaded in the optimized formulation (run 3). The particle size, DI, serum and filtration studies, and drug loading were performed. Like the drug-free microemulsion (DF-ME), it maintained a consistent particle size of <50 nm with uniform particle size distribution (Figure 10A,B) and DI of less than 0.3 (Figure 10C). Both DF-ME and TAC-ME were filtered with a 0.22 µ filter. Both maintained consistent particle sizes within a 10% deviation (Figure 10D). The DF-ME and TAC-ME were studied in 20% FBS in DMEM and incubated at 37 °C, and no significant deviation was observed, indicating their stability while in contact with the biological environment (Figure 10E). The tacrolimus-loaded microemulsion was manufactured in triplicates and showed particle size of <50 nm with uniform particle size distribution, confirming its reproducibility (Figure 10F).

### 3.10. Drug Loading and In Vitro Drug Release Study

**Figure 11:**
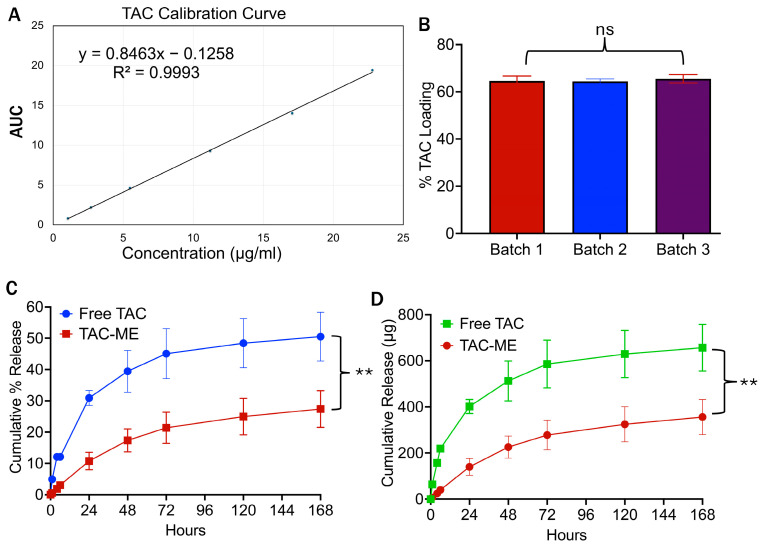
(**A**) The calibration curve is prepared using the reverse phase-HPLC method using a UV wavelength of 210 nm with a retention time of 5.3 min and a flow rate of 0.75 mL/min in triplicates, with LOD = 0.86 and LOQ = 2.6. (**B**) The percentage of tacrolimus loading is quantified using the same technique in three replicates of TAC-loaded microemulsions. (**C**) The cumulative percentage of TAC release from microemulsion and free-tacrolimus solution in release media consisting of 1× PBS and 0.1% Tween 80. (**D**) Cumulative amount of TAC release in μg/mL from microemulsion and free solution. A total of 1300 μg of tacrolimus-loaded microemulsion and an equivalent amount of free tacrolimus solution were loaded into the dialysis bag in triplicates Statistical analysis performed with GraphPad Prism 10 software. Each data point represents an average ± SD (n = 3). Statistical significance is denoted as ** corresponding to the *p*-value < 0.01, and ns indicates not significant.

The standard curve for tacrolimus was developed (Figure 11A) in triplicates using the validated RP-HPLC method. A total of 64.59% of drug loading was achieved. Triplicate tacrolimus-loaded microemulsion was manufactured, and the average drug content for three batches was 1167.10 ± 10.06 µg/mL with 64.84% ± 0.55 of drug loading (Figure 11B).

Moreover, the in vitro release of tacrolimus from free solution and microemulsion was compared at 37 °C. It was observed that the microemulsion provided a release of 27.39 ± 5.86% of tacrolimus, and the free solution released 50% of tacrolimus until 168 h (Figure 11C). The release profile in microemulsion showed a slower and extended-release profile. The cumulative tacrolimus release from microemulsion was 356 µg or 0.3 mg (Figure 11D), indicating a slower release pattern over time. The long-term release study was conducted to confirm that the release was occurring gradually and in a steady manner without showing any burst release.

## 4. Discussion

In the QbD approach, the target product profile with specified CQA ranges is identified as the first step of development for transferring the quality attributes into the final product. The goal of this approach is to deliver quality products to the patient consistently while maintaining the CQAs. Moreover, it helps to identify and establish the relationship between critical parameters (formulation or process) and CQAs [36]. In this study, the QbD approach was followed from scratch to develop tacrolimus-loaded microemulsion for targeting skin inflammation. The QTPP was selected to formulate an oil-in-water microemulsion formulation having a particle size of 30–50 nm and a DI ≤ 0.3 (Table 1). Smaller particle size was included in the QTPP for better drug penetration in the skin. For instance, skin penetration evaluation on finasteride-loaded microemulsion (hydrophobic) having a particle size of less than 80 nm showed a better drug delivery profile than microemulsions with a larger particle size. The results indicated that small particle sizes might better penetrate the skin layers and enhance therapeutic efficacy [37]. For better penetration and enhanced drug delivery profile, particle sizes of 30–50 nm and DI ≤ 0.3 were selected. In the pre-formulation and process screening stage, the initial excipients and process for manufacturing were selected based on the prior work and the literature.

For pre-formulation screening, both long-chain hydrocarbon (safflower oil) and medium-chain hydrocarbon oil (Miglyol 812N) were selected. The oil type and percentage, as well as their required HLB number, are critical factors driving microemulsification. During the screening studies, individual surfactants and their combinations with different percentages were explored. In the pre-formulation studies, the microemulsion formulated with a long hydrocarbon chain in the oil phase was stable and homogeneous when the oil and surfactant ratio (safflower oil: Cremophor EL) was maintained at a 1:4.5 ratio. However, when a combination of the non-ionic surfactant system was utilized (Cremophor EL: PEG 400) in a 1:1 ratio, it resulted in a larger particle size and significantly impacted the stability profile of the microemulsion, resulting in phase separation. The combination of the surfactant system decreased the overall concentration of Cremophor EL by lowering its ratio with oil. The phenomenon may be attributed to the increase in interfacial tension and the inadequate coverage of oil droplets and its ratio of surfactant mixture (Cremophor EL: PEG 400) [31]. Moreover, a single surfactant system was utilized in the following trial (Trial C), reducing the concentration of oil, surfactant, and co-surfactant while keeping the ratio the same. However, only differing the concentration of components in trial A and trial C resulted in a distinct homogeneity profile. Reduction in surfactant and co-surfactant concentrations could minimize the oil and water interfacial coverage and overall stability [38]. As a result, the observed particle size in trial C was higher (339 nm) and eventually became phase-separated. Additionally, keeping the same concentration and ratio of components as in trial A, the type of oil was changed to medium-chain hydrocarbon oil (Miglyol 812N) in trial C. The triglyceride nature and polarity of Miglyol 812N in a 1:4.5 ratio with surfactant (Cremophor EL) resulted in smaller particle sizes (19 nm), attributing to the gel-like crystalline phase [39]. While developing microemulsions, optimizing the type and percentage of oil is critical for determining its homogenous phase. The long-chain fatty acid-based microemulsion is considered advantageous for enhancing the dissolution profile [40]. Moreover, the solubility of tacrolimus in safflower oil is reported to be 0.94 mg/g [41]. Following the results of pre-formulation screening (Table 2), safflower oil was selected as the preferred choice for developing a tacrolimus-loaded microemulsion. Instead of Miglyol 812N, the safflower oil was considered as it showed better homogeneity and a longer colloidal stability profile compared to other trials. Surfactants, such as Cremphor EL and PEG (Polyethylene Glycol) 400, are reported to have tacrolimus solubility profiles of 23.27 mg/mL and 75 mg/mL, respectively [42]. Another study with transcutol as a co-surfactant also shows a better tacrolimus solubility profile compared to other co-surfactants like propylene glycol, soluphor P, Lutrol E 400, etc. [43]. However, the combination of Cremophor EL and PEG 400 in trial C (Table 2) was not explored further for impacting the homogeneity profile of microemulsion. The effect of the aqueous phase was also noted to define the relationship with CQAs, and no significant impact was observed. The trial with PBS as an aqueous phase was selected to maintain the pH (7.4) of the microemulsion [29]. Considering the results from screening trials and supporting details from the literature studies, trial A was selected in Table 2.

To further reduce the particle size, the process screening for the selected trial was performed. The titration method for manufacturing the microemulsion [24] resulted in a particle size greater than 250 nm, which was out of the specified range according to QTPP. Based on the previously published manufacturing approach [28,35], both sonication and microfluidization, in combination with titration, were applied to see the impact on the CQAs. In comparison to sonication, microfluidization with the LM20 microfluidizer^®^ was observed to be efficient in reducing the particle size within the specified range. The LM20 microfluidizer^®^, as a high-pressure technique, has the capability to reduce the particle size through extremely high shear and turbulence [44]. During the process of screening, the particle size was monitored using dynamic light scattering in each pass after microfluidization to understand the impact of shear (Figure 2C). The monitoring of particle size informed us about the process control strategy while manufacturing.

A rigorous quality risk assessment was completed based on FMECA. This approach has been adopted in different studies to identify potential critical parameters [24,33]. In our prior work, artificial oxygen carriers constructed with perfluorocarbon [33] utilized the FMECA approach to identify parameters significantly impacting oxygen delivery efficiency. Moreover, in two studies, it was considered that the surfactant-to-oil ratio, concentration of surfactant, co-surfactant, and oil are high-risk factors and have high RPN values [45], where the stirring rate, stirring time, and temperature were considered as low-risk [46]. The risk assessment matrix above followed these approaches while developing the microemulsion formulation. Based on the high-risk factors, a DoE was applied to study the parameters associated with the formulation and manufacturing process [24]. The D-Optimal design was applied in this study, which is a type of DoE that uses a statistical perspective to create a model that decreases the covariance in the estimated regression coefficients to offer a significant model that best fits a set of data [26,27]. After initial screening and risk assessment, the high-risk factors impacting the CQAs were identified and applied in the DoE. The factors included the percentage of surfactant and co-surfactant, titration rate, and processing pressure in the microfluidizer. These variables have a mix of both continuous and discrete numbers. The responses for these factors are the particle size in diameter, the DI for Day 1 and Day 7, changes in particle size, DI after filtration, and serum studies. The ranges were also set based on the QTPP (Table 1). According to the variables and constraints, the D-optimal DoE only selects an optimal set of data from a wide range of trials and estimates the responses.

For optimizing the microemulsion formulation and process parameters, a set of eight runs was selected for exploring the design space. After the experimental analysis following the selected set of trials from the design space, a fit-model analysis was conducted (Figure 6 and Figure 7) to correlate the selected variables with the CQAs, showing how each factor influenced the response variables. Following the *p*-value for each factor like the percentage of surfactant and co-surfactant, titration rate, and processing pressure in the microfluidizer, the significant effect for each factor was determined. Statistical data from the fit model revealed that the pressure of the microfluidizer was the most significant variable in microemulsion stability for meeting CQA specifications on Day 1 and Day 7, with serum and filtration study based on the *p*-value of <0.05. The high processing pressure (20,000 psi) during microfluidization reduced the particles to relatively smaller sizes, impacting the overall characterization of the microemulsion. While processing at high pressures (20,000 psi), the microemulsion encountered repetitive, intense shear through the interaction chamber in the microfluidizer. The consistent high shear force at 20,000 psi for five passes was effective in reducing the particle size to <50 nm. However, changing the processing pressure to 15,000 psi reduced the level of shear force in the interaction chamber of the microfluidizer, resulting in a particle size of >50 nm (Figure 5A,B). The other variables, such as surfactant and co-surfactant percentage and titration rate, have a *p*-value of >0.05, indicating statistical insignificance in increasing the particle size of >50 nm (Figure 6C,D). However, the variation in the percentage of surfactants and co-surfactants caused a deviation in particle size due to their impact on interfacial surface tension (Figure 5C) [31]. Regardless of adjusting the surfactant and co-surfactant concentration in this design space, the particle size was within the QTPP range. In response, surface Figure 6F,G showed that reducing the processing pressure in microfluidization resulted in particle size out of specification (>50 nm), indicating the edge of failure in this design space. 

Based on the results of DoE trials (Table 6), run 3 was selected and scaled up to 600 mL from 200 mL using the same LM20 microfluidizer^®.^ The scalability profile was assessed, and it maintained the determined CQAs (Figure 9). The control of oil concentration, oil-to-surfactant ratio, surfactant-to-co-surfactant ratio, and process understanding are critical to maintain when scaling up. For successful scale-up, the oil-to-surfactant ratio (1:3.75), surfactant-to-co-surfactant ratio (3.6:1), and processing pressure in microfluidization (20,000 psi) must be controlled to achieve the desired QTPP. The tacrolimus was loaded into microemulsion formulation in the 200 mL scale, and the achieved percentage of drug loading was more than 50%. Tacrolimus is known to have a poor solubility profile in the aqueous phase and is challenging to formulate [47]. However, the successful loading of tacrolimus was achieved in the optimized oil-in-water microemulsion formulation. The optimized formulation achieved colloidal stability and maintained particle size and DI when encountered with filtration and serum stability studies (Figure 10). The targeted smaller particle size (30–50 nm) with a large surface area of microemulsion was achieved to facilitate better penetration in the skin barrier and improve drug permeation in the target site. Efficient drug penetration is an important factor for ensuring bioavailability, making this formulation promising for delivering tacrolimus topically. The drug release study was conducted for a longer time to confirm the slow-release profile in a steady manner without showing any initial burst release [48,49]. This controlled profile might be advantageous for developing the formulation further for extended therapeutic effects and enhancing patient compliance [50]. 

## 5. Conclusions

The adaptation of a QbD approach in this study led to the successful production of microemulsion, which identified a robust process. This novel approach to formulating a tacrolimus-loaded microemulsion with a robust process brings insights into a novel formulation that has broader applications in treating skin inflammatory conditions. This framework accelerated the achievement of the desired QTPP of the microemulsion, meeting all the CQAs. The pre-formulation and process screening stage in the QbD approach offered a ground to start with suitable components and processes for developing the formulation, which is efficient with limited resources and time. Application of the DoE and statistical analysis resulted in microemulsion with increased drug loading. Furthermore, we successfully scaled up the microemulsion from 200 mL to 600 mL, meeting all the CQA specifications. However, the design space can be explored further in a broader range to discover more formulation possibilities. The enhanced tacrolimus-loading microemulsion can be further developed into the thermos-responsive gel [28] or other formulations suitable as topical formulations for local delivery. The concentration of tacrolimus can be adjusted further depending on the target site. Therefore, the QbD approach has made a successful formulation of microemulsion for loading tacrolimus and paved the way for developing it further for broader applications.

## Figures and Tables

**Table 1:** Listed CQAs and their specifications.

Critical Quality Attributes (CQAs)	Specification
Day 1 Microemulsion diameter	30–50 nm
Day 1 DI	≤0.3
Diameter change after filtration	<10%
DI change after filtration	≤0.3
Diameter change after serum stability	<10%
DI change after serum stability	≤0.3
7-day diameter change	<10%
7-day DI	≤0.3
Drug loading	≥50%

**Table 2:** Pre-formulation screening using low-energy method (titration) using different oil phases, different combinations of surfactant and co-surfactant, and aqueous phase.

Trial	Type of Oil	% (*w*/*v*) of Oil	Type of Surfactants	% (*w*/*v*) of Surfactants	Type of Co-Surfactant	% (*w*/*v*) of Co-Surfactant	Type of Aqueous Phase	Particle Size (nm)	DI	Homogeneity (Visual Inspection)
Trial A	Safflower Oil	6%	Cremophor EL	27.5%	Transcutol	7.5%	PBS	254	0.217	Homogeneous
Trial B	Safflower Oil	6%	Cremophor EL: PEG 400 (1:1)	Surfactant A (13.75%) and Surfactant B (13.75%)	Transcutol	7.5%	PBS	711	0.094	Phase separated
Trial C	Safflower Oil	3%	Cremophor EL	13.75%	Transcutol	3.75%	PBS	339	0.275	Phase separated
Trial D	Miglyol 812N	6%	Cremophor EL	27.5%	Transcutol	7.5%	PBS	19.26	0.043	(Gel-like consistency)
Trial E	Safflower Oil	6%	Cremophor EL	27.5%	Transcutol	7.5%	Water	264.4	0.219	Homogeneous

**Table 3:** Description of different levels of risk priority number values (starting from too low to too high) for severity, frequency of occurrence, and detectability.

**Scale**	1	2	3	4	5
**Level**	Too Low	Low	Medium	High	Too High
**Severity (S)**	No consequences to batch quality	Minor deviations that cause insignificant consequences to batch quality and batch recoverable	Consequences that require actions occur, and the batch is recoverable	Major deviations that cause significant consequences to batch quality and difficulty in batch recovery	Total batch loss
**Frequency of occurrence (O)**	Risks that do not happen	Risks that happen rarely	Risks that happen sporadically	Risks that occur frequently	Risks that happen most of the time
**Detectability (D)**	Readily detected	Moderately detected	Detected but not always or not promptly	Difficult to detect	Not detectable within the current manufacturing operation

**Table 4:** A risk assessment matrix of formulation parameters and process parameters based on effect on CQA of microemulsion. This matrix has been prepared by calculating the RPN value for each risk factor in terms of their effect on different quality attributes of microemulsion.

CQAs	Risk Factors							
	% (*w*/*v*) of Oil	% (*w*/*v*) of Surfactant	% (*w*/*v*) of Co-Surfactant	Water Addition Rate (mL/min)	Stirring Time (h)	Stirring Rate (rpm)	Pressure of Microfluidizer (psi)	Temperature
Particle Size/ Droplet Diameter	High	High	High	High	Low	Low	High	Low
Dispersity Index	High	High	High	High	Low	Low	High	Low
Drug Loading	High	High	High	High	Low	Low	High	Low
Homogeneity	High	High	High	High	Medium	Medium	High	Low

**Table 5:** Identification of the high-risk factors based on risk priority numbers (RPN), their method of failure, and list of CQAs that are impacted along with the cause of failure. Severity, occurrence, and detectability are presented as S, O, and D, respectively.

S	O	D	RPN	Method of Failure	CQA Impacted	Cause of Failure
5	3	5	75	Droplet diameter (>50 nm or <30 nm) DI (>0.3) Drug loading (<50%) Inhomogeneity	Droplet diameter DI Drug loading Homogeneity	Too fast or too slow titration rate
4	4	5	80	Droplet diameter (>50 nm or <30 nm) DI (>0.3) Drug loading (<50%) Inhomogeneity	Droplet diameter DI Drug loading Homogeneity	Too high or too low percentage of oil
5	3	5	75	Droplet diameter (>50 nm or <30 nm) DI (>0.3) Drug loading (<50%) Inhomogeneity	Droplet diameter DI Drug loading Homogeneity	Too high or too low percentage of surfactant
5	3	5	75	Droplet diameter (>50 nm or < 30 nm) DI (>0.3) Drug loading (<90% or >100%) Inhomogeneity	Droplet diameter DI Drug loading Homogeneity	Too high or too low percentage of cosurfactant
5	3	5	75	Droplet diameter (>50 nm or < 30 nm)) DI (>0.3) Drug loading (<50%) Inhomogeneity	Droplet diameter DI Drug loading Homogeneity	Too high or too low pressure in Microfluidizer

**Table 6:** The summary of the results from D-optimal design of experiments.

Run	Day 1 Diameter (30–50 nm)	Day 1 DI (<0.30)	Diameter Change After Filtration (<10%)	DI After Filtration (<0.3)	Diameter Change After Serum Stability Test (<10%)	DI After Serum Stability Test DI (<0.3)	Day 7 Diameter Change (<10%)	Day-7 DI (<0.30)
1	39.89	0.21	−8.09	0.197	**13.79**	**0.302**	0.23	0.206
2	**50.79**	0.24	2.66	0.220	−1.9	**0.336**	0.28	0.236
**3**	**45.02**	**0.22**	**8.6**	**0.191**	**−3.09**	**0.287**	**1.91**	0.212
4	**54.78**	0.23	3.53	0.228	−0.3	**0.385**	1.96	0.238
5	41.32	0.23	3.8	0.204	1.59	**0.328**	1.28	0.220
6	43.15	0.20	3.47	0.194	**13.18**	**0.362**	−3.38	0.228
7	**50.01**	0.22	3.64	0.200	3.65	**0.315**	1.03	0.224
8	**52.95**	0.22	2.96	0.200	1.86	**0.315**	−2.68	0.221

% Diameter change = [(Initial Diameter − Diameter after each CQA study)/Initial diameter] × 100. (−) sign indicates the increase in size after filtration and serum stability, (_) Underscore indicates the value is out of specification. The highlighted row indicates the run that meets the CQA specifications across all assessments.

## Data Availability

Data can be made available for non-commercial uses and per reasonable request made to the corresponding author at janjicj@duq.edu.

## Supplementary Materials

The following supporting information can be downloaded at https://www.mdpi.com/article/10.3390/pharmaceutics16121487/s1, Figure S1: Particle size distribution in each passage after microfluidization starting from lower (15,000 psi) to higher pressure (20,000 psi); Figure S2: The residual vs. leverage plots to describe the relationship between individual variables and particle size as response factors in Day 7 diameter in microemulsion trials; Figure S3: The actual vs. predicted value of the model for the predicting particle after serum and filtration study.
