# Supplementary material for: Quality by Design (QbD)-Driven Development and Optimization of Tacrolimus-Loaded Microemulsion for the Treatment of Skin Inflammation
Source: Pharmaceutics. 2024 Nov 21;16(12):1487. doi: 10.3390/pharmaceutics16121487 (PMC11678404; doi:10.3390/pharmaceutics16121487)
Supplement: Supplementary file 1 [file pharmaceutics-16-01487-s001.zip › pharmaceutics-3269462-supplementary.pdf]

SUPPLEMENTAL MATERIALS

Quality by Design (QbD)-Driven Development and Optimization of Tacrolimus-Loaded Microemulsion for the Treatment of Skin Inflammation

Sanjida Ahmed Srishti, Paromita Paul Pinky, Ryan Taylor, Jacob Guess, Natasha Karlik and Jelena M. Janjic *

School of Pharmacy, Graduate School of Pharmaceutical Sciences, Duquesne University, Pittsburgh, PA 15282, USA; srishtis@duq.edu (S.A.S.); pinkyp@duq.edu (P.P.P.); taylorr5@duq.edu (R.T.); guessj@duq.edu (J.G.); karlikn@duq.edu (N.K.)

* Correspondence: janjicj@duq.edu

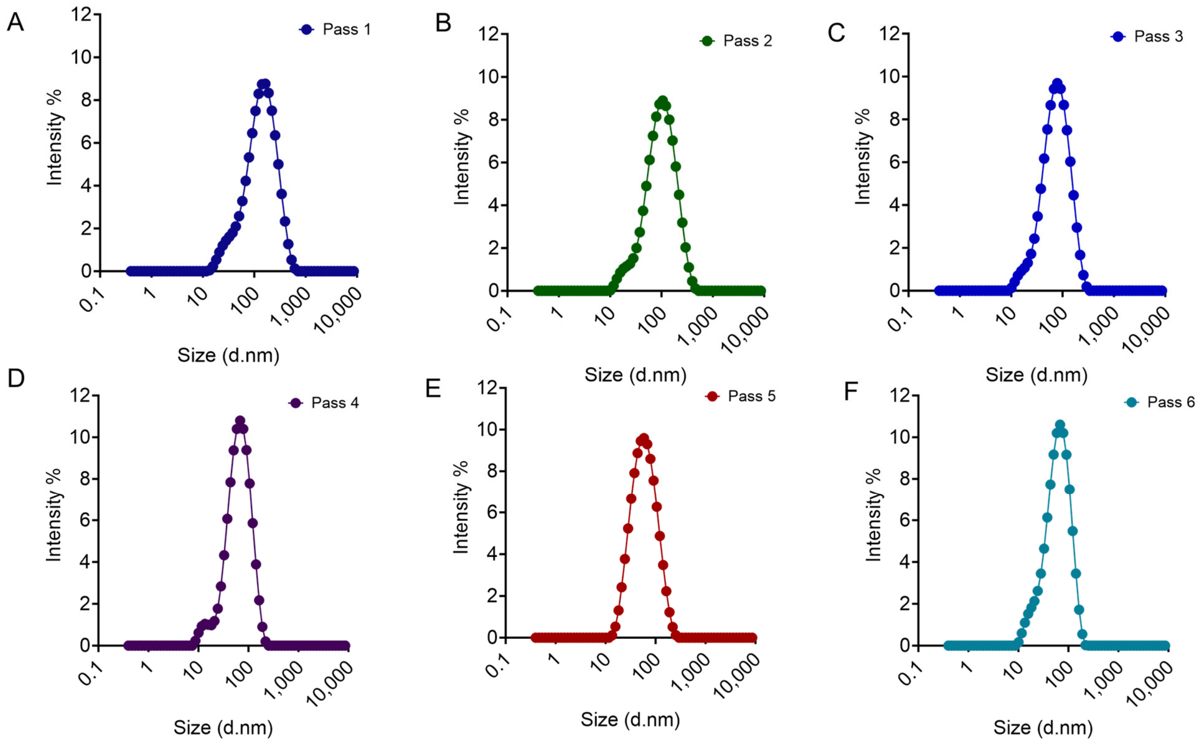

Supplementary Figure S1. The figure (**A–F**) shows particle size distribution in each passage after microfluidization starting from lower (15,000 PSI) to higher pressure (20,000 PSI). After 5 passage (**E**) the size of microemulsion decreased confirming uniform distribution. the particle size distribution observed to be more uniform compared to the 6 passage (**F**) and 5 passage during microfluidization was selected based on that.

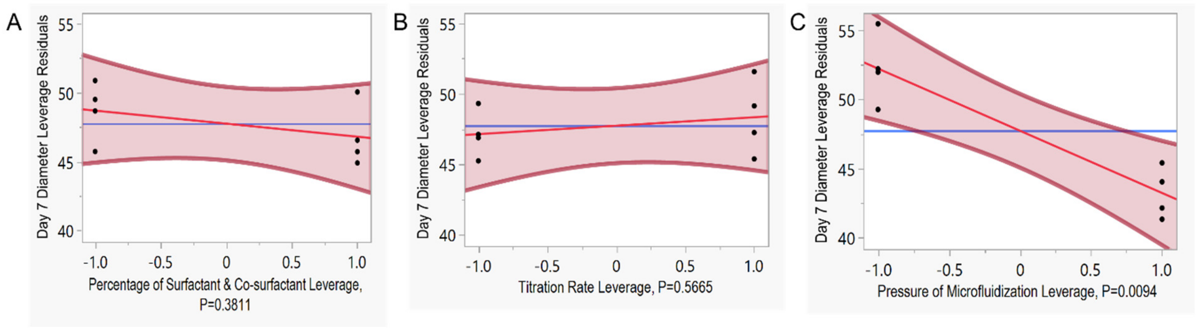

Supplementary Figure S2. The plots (**A**, **B** & **C**) demonstrate residual vs leverage to describe the relationship between individual variables and particle size as response factors in Day 7 diameter in microemulsion trials. The residual vs leverage plot represents the individual main effects of independent variables (**A**. Percentage of surfactant & co-surfactant, **B**. titration rate and **C**. pressure of microfluidization) for Day 7 indicating that percentage of surfactant and co-surfactant, titration rate have no significant impact on Day 7 diameter of microemulsion ($p > 0.05$). However, the pressure of microfluidization has a significant ($p < 0.05$) impact on the day 7 diameter of microemulsion.

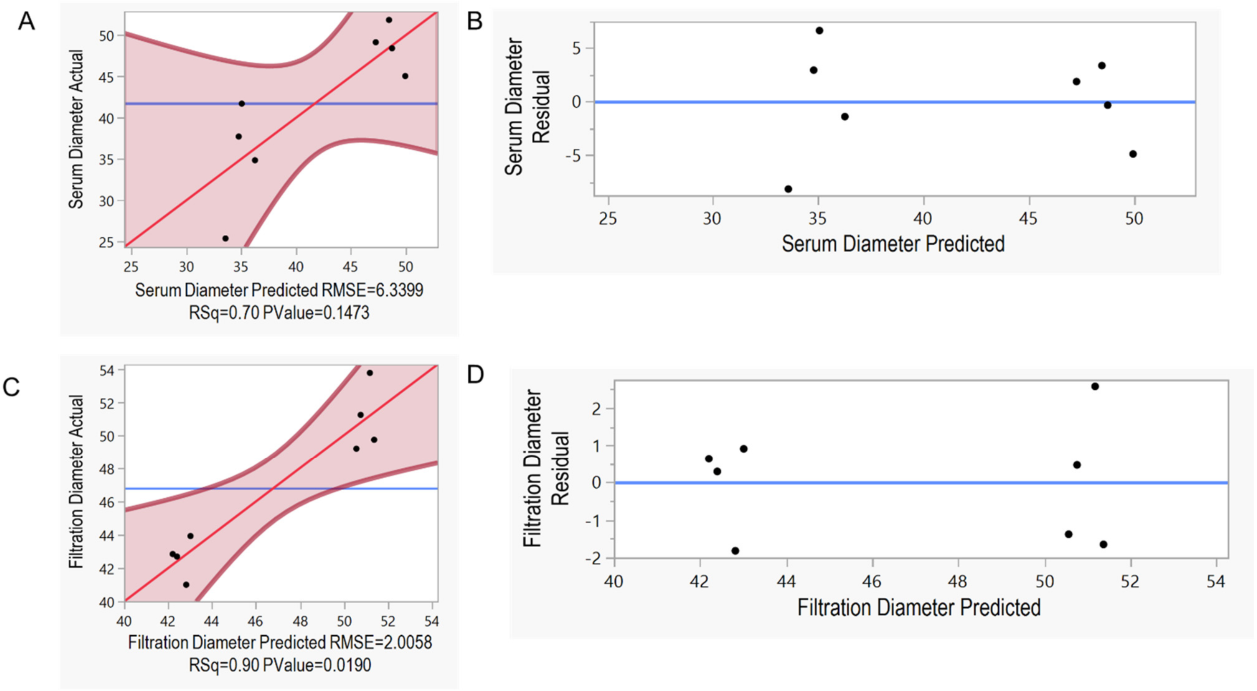

Supplementary Figure S3. The plot (**A**) illustrates the actual vs predicted value of the model suggesting that, the parameters involved in the model is not significant for predicting the diameter after serum studies as the $p > 0.05$, however, the residual vs predicted plot (**B**) shows a random distribution of points showing an independent behaviour. Plot (**C**) shows that parameters involved in the model is statistically significant ($p < 0.05$) for prediction in diameter after filtration studies and demonstrates a random pattern in residual vs predicted plot (**D**).
